# Patients with *ASPSCR1-TFE3* fusion achieve better response to ICI based combination therapy among *TFE3*-rearranged renal cell carcinoma

**DOI:** 10.1186/s12943-024-02044-5

**Published:** 2024-06-26

**Authors:** Junjie Zhao, Yanfeng Tang, Xu Hu, Xiaoxue Yin, Yuntian Chen, Junru Chen, Haoyang Liu, Haolin Liu, Jiayu Liang, Xingming Zhang, Jinge Zhao, Sha Zhu, Yuchao Ni, Zhipeng Wang, Jindong Dai, Zilin Wang, Yaowen Zhang, Jin Yao, Ni Chen, Pengfei Shen, Zhenhua H. Liu, Hao Zeng, Guangxi X. Sun

**Affiliations:** 1 Department of Urology, Institute of Urology, Sichuan Clinical Research Center for kidney and urologic diseases, West China Hospital, Sichuan University, No.37 Guoxue Alley, Wuhou District, Chengdu, Sichuan Province China; 2 Department of Pathology, West China Hospital, Sichuan University, Chengdu, China; 3 Department of Radiology, West China Hospital, Sichuan University, Chengdu, China

**Keywords:** *TFE3*-rRCC, Fusion partner, *ASPSCR1*, Immune checkpoint inhibitor, Combination therapy, Molecular correlates, ECM, Angiogenesis, Immune microenvironment

## Abstract

**Background:**

*TFE3*-rearranged renal cell carcinoma (*TFE3*-rRCC) is a rare but highly heterogeneous renal cell carcinoma (RCC) entity, of which the clinical treatment landscape is largely undefined. This study aims to evaluate and compare the efficacy of different systemic treatments and further explore the molecular correlates.

**Methods:**

Thirty-eight patients with metastatic *TFE3*-rRCC were enrolled. Main outcomes included progression-free survival (PFS), overall survival, objective response rate (ORR) and disease control rate. RNA sequencing was performed on 32 tumors.

**Results:**

Patients receiving first-line immune checkpoint inhibitor (ICI) based combination therapy achieved longer PFS than those treated without ICI (median PFS: 11.5 vs. 5.1 months, *P* = 0.098). After stratification of fusion partners, the superior efficacy of first-line ICI based combination therapy was predominantly observed in *ASPSCR1-TFE3* rRCC (median PFS: not reached vs. 6.5 months, *P* = 0.01; ORR: 67.5% vs. 10.0%, *P* = 0.019), but almost not in non-*ASPSCR1-TFE3* rRCC. Transcriptomic data revealed enrichment of ECM and collagen-related signaling in *ASPSCR1-TFE3* rRCC, which might interfere with the potential efficacy of anti-angiogenic monotherapy. Whereas angiogenesis and immune activities were exclusively enriched in *ASPSCR1-TFE3* rRCC and promised the better clinical outcomes with ICI plus tyrosine kinase inhibitor combination therapy.

**Conclusions:**

The current study represents the largest cohort comparing treatment outcomes and investigating molecular correlates of metastatic *TFE3*-rRCC based on fusion partner stratification. ICI based combination therapy could serve as an effective first-line treatment option for metastatic *ASPSCR1-TFE3* rRCC patients. Regarding with other fusion subtypes, further investigations should be performed to explore the molecular mechanisms to propose pointed therapeutic strategy accordingly.

**Supplementary Information:**

The online version contains supplementary material available at 10.1186/s12943-024-02044-5.

## Introduction

*TFE3*-rearranged renal cell carcinoma (*TFE3*-rRCC), characterized by Xp11.2 translocations involving *TFE3*, is a rare variant of renal cell carcinoma (RCC) [1]. Unlike other more common subtypes of RCC, *TFE3*-rRCC has a distinct demographic profile, with a younger age at diagnosis, an advanced stage at presentation, and a female predominance [2]. Due to the rarity of *TFE3*-rRCC, the understanding of the underlying mechanisms is currently poor, and the clinical treatment strategy in *TFE3*-rRCC is also largely undefined. To date, more than twenty *TFE3* gene fusion partners have been identified, including *ASPSCR1*, *SFPQ*, *NONO*, *PRCC*, *RBM10*, *MED15*, etc. The diversity of fusion partners and chromosome structures affects biological behaviors and leads to the high heterogeneity of *TFE3*-rRCC, both morphologically and clinically [3]. In clinical practice, diagnosis with *TFE3*-rRCC is mainly depended on IHC staining and FISH with break-apart probes, losing exact information about fusion partner. What’s worse, being lack of effective therapy, patients with metastases had to be treated with established standard therapies developed for clear cell RCC (ccRCC), including tyrosine kinase inhibitors (TKI), mammalian target of rapamycin inhibitors (mTORi) and immune checkpoint inhibitors (ICI). So far, although there exists case reports and retrospective studies reporting some responses to these therapies, outcomes have been variable between series [4–7]. Hence, the optimal therapy for *TFE3*-rRCC, especially with different fusion partner genes, remains to be determined.

Based on our previous transcriptomic analysis, *TFE3*-rRCC could be classified into 5 molecular clusters, suggesting that patients with *TFE3*-rRCC theoretically needed to be differentially treated [1]. Nevertheless, no studies have yet compared responses of patients based on different fusion partners to systemic treatments. Therefore, we performed a single-center retrospective analysis in metastatic *TFE3*-rRCC patients with definite fusion partner genes based on RNA-seq, evaluating and comparing the efficacy of different systemic treatments and further exploring the molecular correlates.

## Results

### Patient characteristics

**Fig. 1:**
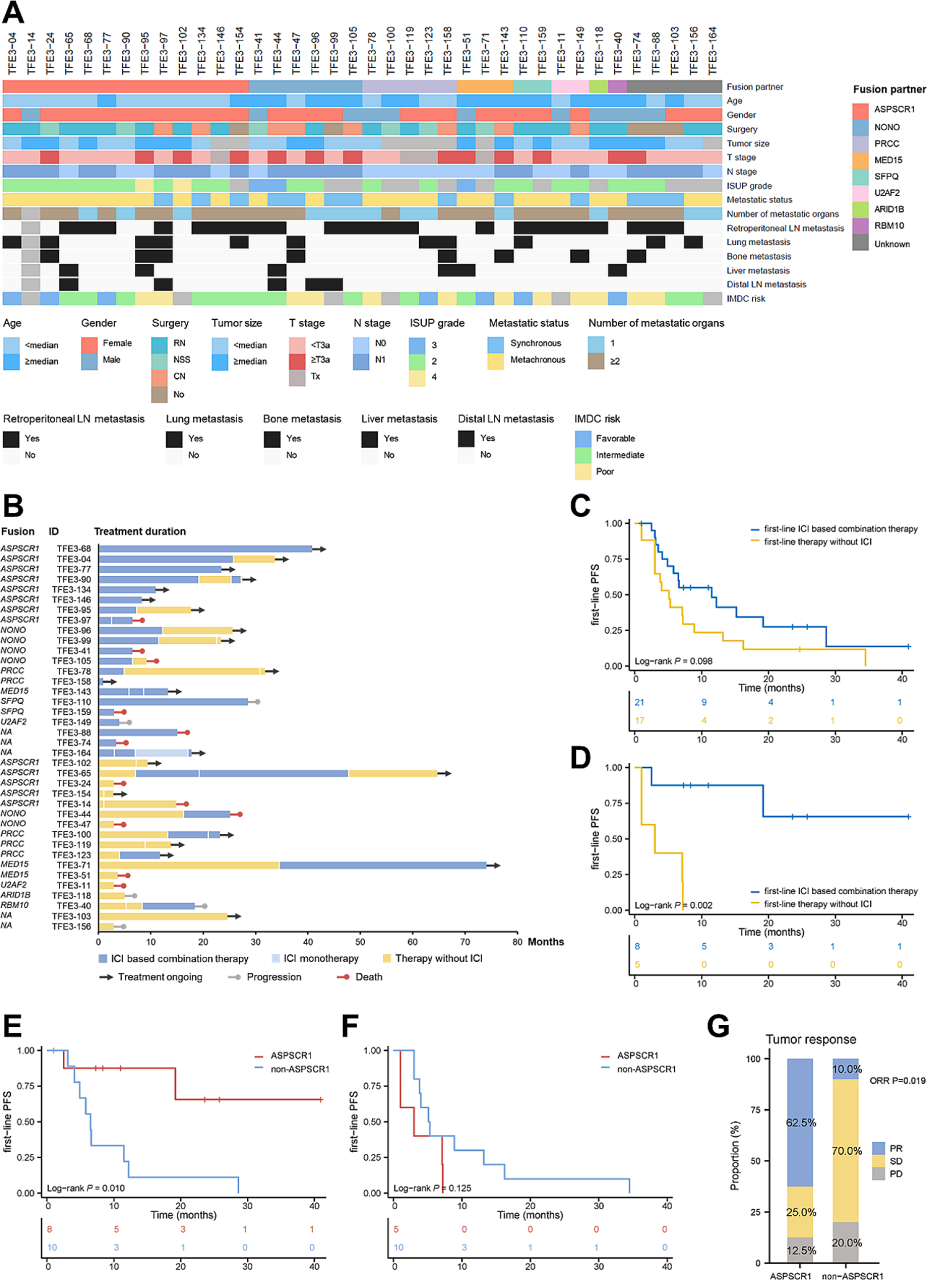
Characteristics and treatment outcomes of patients with metastatic *TFE3*-rRCC. **A** Heatmap showing clinicopathologic characteristics of patients with metastatic *TFE3*-rRCC patients (*n* = 38). **B** Swimmer plot showing the treatment response and duration of each patient receiving systemic treatments. **C** First-line PFS between patients receiving ICI based combination therapy and therapy without ICI at first-line. **D** First-line PFS between *ASPSCR1-TFE3* rRCC patients receiving ICI based combination therapy and therapy without ICI at first-line. **E** First-line PFS between patients with *ASPSCR1-TFE3* rRCC and non-*ASPSCR1-TFE3* rRCC when receiving first-line ICI based combination therapy. **F** First-line PFS between patients with *ASPSCR1-TFE3* rRCC and non-*ASPSCR1-TFE3* rRCC when receiving first-line therapy without ICI. **G** Tumor response between patients with *ASPSCR1-TFE3* rRCC and non-*ASPSCR1-TFE3* rRCC when receiving first-line ICI based combination therapy

A total of 38 patients with metastatic *TFE3*-rRCC from our RCC database were enrolled in the study from January 2011 to February 2023. The median follow-up time was 28.8 months (range: 3.7-153.2). The baseline characteristics of the included patients are summarized in Suppl. Table 1 and Fig. 1A. The median age at initial diagnosis was 36 years (range: 11–70 years) and the male-to-female ratio was 1:2.5. Regarding nephrectomy, 50.0% (19/38), 15.8% (6/38) and 21.1% (8/38) of patients underwent radical nephrectomy (RN), nephron-sparing surgery (NSS) and cytoreductive nephrectomy (CN), respectively. The median size of tumors was 5.7 cm (range: 2.2–19.4 cm) and 60.5% (23/38) tumors had an ISUP grade ≥ 3. Collectively, the fusion partners were identified in 33/38 (86.8%) patients, including *ASPSCR1-TFE3* (*n* = 13), *NONO-TFE3* (*n* = 6), *PRCC-TFE3* (*n* = 5), *MED15-TFE3* (*n* = 3), *SFPQ-TFE3* (*n* = 2), *U2AF2-TFE3* (*n* = 2), *RBM10-TFE3* (*n* = 1), *ARID1B-TFE3* (*n* = 1).

Within the cohort, 65.8% (25/38) patients had metachronous metastases. Twenty-four (63.2%) patients developed metastasis in more than one organ. Involvement of retroperitoneal lymph node was the most common site of metastasis at the time of diagnosis of metastatic disease (60.5%). Lung, bone, liver and distal lymph node metastases were each observed at diagnosis of metastatic disease in 28.9%, 21.1%, 15.8%, 13.2% of cases, respectively. Among 32 patients with complete clinical data to evaluate IMDC risk, 78.1% (25/32) patients were stratified into intermediate/poor-risk group.

### Systemic treatment outcomes of metastatic *TFE3*-rRCC 

All the 38 patients received systemic treatment. The details of systemic therapies for individual patients on each treatment line are shown in Fig. 1B. For the first-line treatment, 21 patients received ICI based combination therapy, including ICI plus TKI (*n* = 19) (sitilimab plus axitinib, *n* = 9; toripalimab plus axitinib, *n* = 4; tislelizumab plus axitinib, *n* = 2; pembrolizumab plus sunitinib, *n* = 1) and ICI plus TKI plus mTORi (*n* = 2) (toripalimab plus axitinib plus everolimus, *n* = 2). The other 17 patients received therapy without ICI, including TKI monotherapy (*n* = 13) (axitinib, *n* = 6; sunitinib, *n* = 5; sorafenib, *n* = 2) and TKI plus mTORi (*n* = 4) (axitinib plus everolimus, *n* = 3; lenvatinib plus everolimus, *n* = 1).

For the first-line setting, the median PFS was 6.6 (95% CI: 4.3–8.9) months, and the median OS was not reached (Suppl. Figure 1 A, 1B). All patients were evaluable for the best tumor response by RECIST 1.1. Patients receiving ICI based combination therapy had a higher ORR (7/21, 33.3%) than those treated without ICI (3/17, 17.6%) (*P* = 0.275), as well as DCR (16/21, 76.2% vs. 8/17, 47.1%, *P* = 0.064) (Suppl. Figure 2 A). Compared to patients treated without ICI at first-line, those receiving ICI based combination therapy at first-line had longer PFS (median PFS: 11.5 vs. 5.1 months, *P* = 0.098), although without statistical significance **(**Fig. 1C**)**. Regarding OS, there was no significant difference between the two groups (median OS: not reached vs. 27.4 months, *P* = 0.593) (Suppl. Figure 2B). However, after comparing patients receiving first or subsequent-line ICI based combination therapy with those who never received ICI during their treatment history, we found that the former had longer OS (median OS: not reached vs. 15.2 months, *P* = 0.045) (Suppl. Figure 2 C).

Treatment-related adverse events (AEs) of any grade occurred during first-line treatment are summarized in Suppl. Table 2. The most common AEs were palmar-plantar erthrodysesthesia syndrome (20/38, 52.6%), proteinuria (15/38, 39.5%), increased blood creatinine (14/38, 36.8%). Most AEs were grade 1/2, AEs of grade ≥ 3 occurred in 9 (23.7%) patients. For patients receiving ICI based combination therapy and patients treated without ICI at first-line treatment, AEs of grade ≥ 3 occurred in 6 (28.6%) and 3 (17.6%) patients, respectively.

### ICI based combination therapy improved prognosis in *ASPSCR1-TFE3* rRCC

Subgroup analysis was further performed to identify patients in whom first-line ICI based combination therapy could effectively improve PFS (Suppl. Figure 3). The results demonstrated that, first-line ICI based combination therapy significantly prolonged PFS compared with therapy without ICI in patients younger than median age (PFS HR: 0.177, 95% CI: 0.045–0.705, *P* = 0.014), patients with ISUP grade ≥ 3 (PFS HR: 0.183, 95% CI: 0.058–0.578, *P* = 0.004), and patients having metachronous disease (PFS HR: 0.349, 95% CI: 0.137–0.894, *P* = 0.028). Moreover, we observed that patients with *ASPSCR1-TFE3* fusion significantly benefited from ICI based combination therapy (PFS HR: 0.068, 95% CI: 0.008–0.609, *P* = 0.016), whereas no improvement in PFS was observed in patients with other fusions (PFS HR: 1.063, 95% CI: 0.416–2.714, *P* = 0.898). Kaplan-Meier curves illustrated that, among patients with *ASPSCR1-TFE3* fusion, those receiving ICI based combination therapy achieved longer PFS than those treated with therapy without ICI (median PFS: not reached vs. 3.0 months, *P* = 0.002) **(**Fig. 1D**)**.

Univariate and multivariate cox regression analysis was further performed to identify prognostic factors that can determine the response to ICI. In the univariate analysis, we found that metastatic status (synchronous vs. metachronous), involvement of bone metastasis and *ASPSCR1-TFE3* fusion were significantly associated with treatment outcomes (Suppl. Table 3). Kaplan-Meier curves demonstrated that patients with *ASPSCR1-TFE3* fusion had longer PFS than those with other fusions when receiving ICI based combination therapy at first-line (median PFS: not reached vs. 6.5 months, *P* = 0.01) (Fig. 1E, Suppl. Table 4). In the contrary, patients with *ASPSCR1-TFE3* fusion could not benefit from first-line therapy without ICI (median PFS: 3.0 vs. 5.1 months, *P* = 0.125) **(**Fig. 1F, Suppl. Table 4). In the multivariate analysis, only *ASPSCR1-TFE3* fusion remained as the only independent prognostic factor for PFS in patients receiving first-line ICI based combination therapy (Suppl. Table 3). Regarding tumor response, ORR was 62.5% (5/8) in *ASPSCR1-TFE3* rRCC patients, much higher than that in other *TFE3*-rRCC subtypes (10.0%, 1/10) (*P* = 0.019) **(**Fig. 1G, Suppl. Table 4). We also observed that patients with *ASPSCR1-TFE3* fusion experienced longer OS compared to those with other fusions when receiving ICI based combination therapy at first-line or subsequent line. However, the difference did not reach statistical significance. (Suppl. Figure 4 A, 4B). Meanwhile, we have utilized data from the IMmotion151 cohort to corroborate our findings (Suppl. Table 5). Although the cohort included only 5 patients with metastatic *TFE3*-rRCC received ICI plus TKI combination therapy in the IMmotion151 cohort, it is notable that the sole responder (PR) was an *ASPSCR1-TFE3* rRCC case, which also exhibited the best PFS. This outcome provides some validation of our findings.

### Transcriptome features of *ASPSCR1-TFE3* rRCC correlated with response to systemic treatment

We then attempted to figure out why patients with *ASPSCR1-TFE3* fusion exhibited better response to ICI based combination therapy than other fusions through transcriptome analysis. RNA-seq was performed on 32 tumors, of which 31 had information of fusion partners. Additionally, we derived data from the IMmotion151 trial to validate findings from our cohort, and partner genes were identified in 10 out of 12 patients with *TFE3* fusion (Suppl. Table 5) [8].

**Fig. 2:**
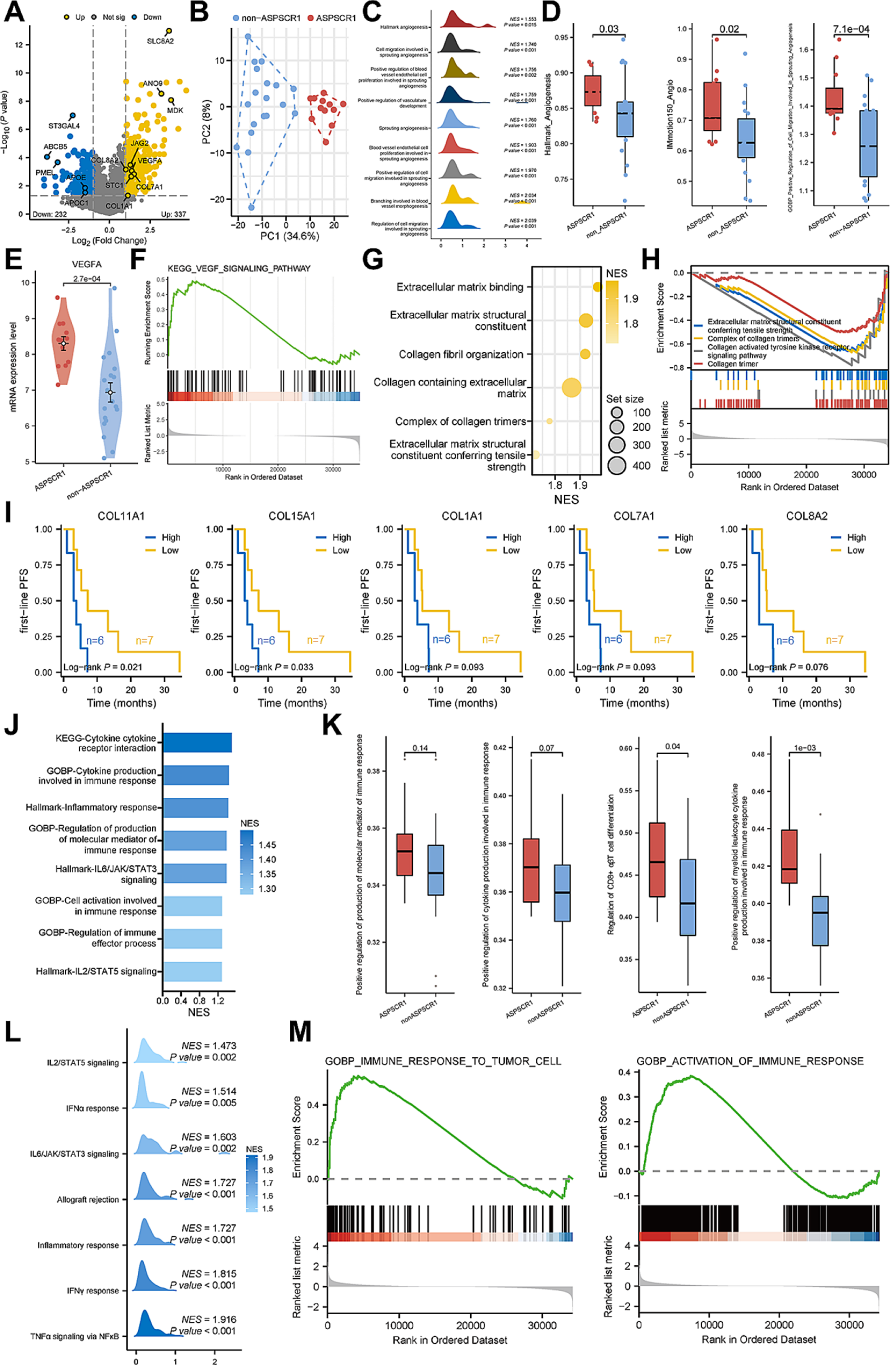
Transcriptomic landscape between *ASPSCR1-TFE3* rRCC and non-*ASPSCR1-TFE3* rRCC. **A** Differentially expressed genes between *ASPSCR1-TFE3* rRCC and non-*ASPSCR1-TFE3* rRCC. **B** Principal component analysis for the expression profiles between *ASPSCR1-TFE3* rRCC and non-*ASPSCR1-TFE3* rRCC. **C** Enrichment of angiogenesis signaling pathways in *ASPSCR1-TFE3* rRCC revealed by gene set enrichment analysis (GSEA). **D** Scores of angiogenesis signaling pathways between *ASPSCR1-TFE3* rRCC and non-*ASPSCR1-TFE3*-rRCC by single sample GSEA (ssGSEA). **E** The mRNA expression level of *VEGFA* between *ASPSCR1-TFE3* rRCC and non-*ASPSCR1-TFE3* rRCC. **F** Enrichment of VEGF signaling pathway in *ASPSCR1-TFE3* rRCC revealed by GSEA. **G** Enrichment of ECM and collagen-related pathways in *ASPSCR1-TFE3* rRCC revealed by GSEA. **H** Down-regulation of ECM and collagen-related pathways in responders of all patients receiving first-line therapy without ICI, irrespectively of fusion type. **I** First-line PFS between patients with high and low expression of collagen-related genes when receiving first-line therapy without ICI, irrespectively of fusion type. **J** Enrichment of immune-related signaling pathways in *ASPSCR1-TFE3* rRCC revealed by GSEA. **K** Scores of immune-related signaling pathways between *ASPSCR1-TFE3* rRCC and non-*ASPSCR1-TFE3* rRCC by ssGSEA. **L** Enrichment of immune-related signaling pathways in *ASPSCR1-TFE3* rRCC revealed by GSEA in IMmotion151 cohort. **M** Enrichment of immune-related signaling pathways in responders receiving first-line ICI based combination therapy

Analysis of differentially expressed genes (DEGs) identified a total of 337 up-regulated and 232 down-regulated genes in *ASPSCR1-TFE3* rRCC compared to non-*ASPSCR1-TFE3* rRCC **(**Fig. 2A**)**. Principal component analysis further revealed that *ASPSCR1-TFE3* rRCC and non-*ASPSCR1-TFE3* rRCC harbored distinct transcriptomic landscapes **(**Fig. 2B**)**. We firstly observed enrichment of multiple pathways involved in angiogenesis by gene set enrichment analysis (GSEA) in both our cohort and the IMmotion151 cohort **(**Fig. 2C, Suppl. Figure 5 A). Scores calculated using single sample GSEA (ssGSEA) further confirmed a higher activity of angiogenesis in *ASPSCR1-TFE3* rRCC (*P* < 0.05) **(**Fig. 2D**)**. As a central regulator of neovasculature and common target of numerous anti-angiogenic agents, the expression level of *VEGFA* at mRNA level was higher in *ASPSCR1-TFE3* rRCC (*P* < 0.001) **(**Fig. 2E, Suppl. Figure 5B). In addition, enrichment of VEGF signaling was also observed in *ASPSCR1-TFE3* rRCC, suggesting the probable favorable response to anti-angiogenic therapy **(**Fig. 2F**)**. Obviously, omics features and clinical practice was inconsistent. In the present cohort, 5 patients with *ASPSCR1-TFE3* fusion could not benefit from TKI based therapy **(**Fig. 1F, Suppl. Table 4).

At the same time, in *ASPSCR1-TFE3* rRCC, we also found enrichment of pathways involved in extracellular matrix (ECM) and collagen by GSEA **(**Fig. 2G, Suppl. Figure 6). Conversely, down-regulation of these pathways were observed in responders (PR), compared to non-responders (SD + PD), in all the patients who received first-line therapy without ICI, irrespectively of fusion types **(**Fig. 2H**)**. Moreover, high expression of several collagen-related genes indicated shorter PFS in all the patients receiving therapy without ICI **(**Fig. 2I**)**. These results demonstrated that ECM and collagen-related pathways enriched in *ASPSCR1-TFE3* rRCC might interfere with the potential efficacy of anti-angiogenic therapy. To our interest, positive correlations were observed between collagen-related genes, ECM and collagen-related signaling activity and expression of immune checkpoints (*CD274*, *PDCD1*), indicating the application of ICI (Suppl. Figure 7).

The immune microenvironment between *TFE3*-rRCC with *ASPSCR1-TFE3* and other fusion partners was then explored. GSEA and ssGSEA both indicated enrichment of multiple immune-related pathways in *ASPSCR1-TFE3* rRCC, which was further verified by data from the IMmotion151 trial **(**Fig. 2A-C**)**. Similarly, up-regulation of immune-related pathways were also observed in responders receiving ICI based combination therapy **(**Fig. 2D**)**. In addition, we calculated and compared the infiltration level of immune cells between *ASPSCR1-TFE3* rRCC and non-*ASPSCR1-TFE3* rRCC using CIBERSORT algorithm (Suppl. Figure 8 A). Our focus then shifted to T cells due to their vital roles in tumor immunity. We observed that T cell levels are comparable between *ASPSCR1-TFE3* rRCC and non-*ASPSCR1-TFE3* rRCC (Suppl. Figure 8B). we also evaluated the expression of several immune checkpoint molecules, finding that the *ASPSCR1-TFE3* rRCC exhibited lower *CTLA4* expression level than the non-*ASPSCR1-TFE3* rRCC (*P* = 0.014) (Suppl. Figure 9). The results indicated a relative more inflamed immune microenvironment in *ASPSCR1-TFE3* rRCC, which might explain why these patients could benefit from ICI based combination therapy.

## Discussion

*TFE3*-rRCC is a rare but highly heterogeneous RCC entity, of which the clinical treatment landscape is largely undefined currently. Thus, the exploration of effective treatments remains an unmet need for this lethal disease. To our knowledge, this study represents the largest cohort investigating treatment outcomes and molecular correlates of metastatic *TFE3*-rRCC. Stratified by fusion partners using RNA-seq, it is the first time for us to reveal the association of therapeutic efficacy with different fusion partners among metastatic *TFE3*-rRCC. Accurately speaking, the superior efficacy of first-line ICI based combination therapy was predominately observed in *ASPSCR1-TFE3* rRCC but almost not in non-*ASPSCR1*-*TFE3* rRCC. Analysis with transcriptomic data further revealed that enrichment of ECM and collagen-related signaling might be involved in interfering with the potential efficacy of anti-angiogenic therapy. However, exclusively enrichment of angiogenesis and immune activities within *ASPSCR1-TFE3* rRCC promised the better clinical outcomes with ICI plus TKI combination therapy.

The lack of standard treatment for patients with metastatic *TFE3*-rRCC is due mainly to its rarity, and the poor understanding of its underlying mechanisms. Early in 2021, we performed unsupervised transcriptomic analysis of 63 untreated *TFE3*-rRCCs and revealed five molecular clusters with distinct transcriptomic landscape [1]. Surprisingly, tumors with *ASPSCR1-TFE3* fusion were exclusively classified together, exhibiting high expression of angiogenesis/stroma/proliferation gene signatures. In contrast to *ASPSCR1-TFE3* rRCC, non-*ASPSCR1-TFE3* rRCC was featured by decreased angiogenesis gene signatures. Differential transcriptomic features among *TFE3*-rRCC with various fusion partners implied diversity with therapeutic strategies to substantially improve clinical outcomes. While almost all of studies with rRCC did not distinguish every fusion partner from each other. Efficacy evaluation could not get a definite conclusion based on types of fusion partner. Moreover, ICI plus TKI was mostly used as subsequent line treatment in these studies, while its therapeutic value as first-line treatment of *TFE3*-rRCC remains unknown. In our study, we initially observed the superior efficacy of first-line ICI based combination therapy in patients with *ASPSCR1-TFE3* rRCC.

Totally speaking, omics analysis for the whole *TFE3*-rRCC demonstrated inactivation of angiogenesis, and several clinical explorations also confirmed the limited efficacy with TKI monotherapy [1]. While comparing the transcriptome features of different fusions, *ASPSCR1*-TFE3 had higher enrichment of angiogenetic activity than other fusion partners, suggesting potential better response to anti-angiogenetic therapy. Unfortunately, in the present study, we still did not observe favorable response as we expected. To our interest, we observed up-regulation of ECM and collagen pathways in non-responders receiving therapy with ICI. ECM network is established by multiple components, among which collagen plays the main role. Studies have demonstrated that there exists interplay between ECM remodeling and angiogenesis, for that ECM creates suitable environment that promotes endothelial cell proliferation, migration, and ultimately, angiogenesis [9]. Nguyen et al. previously reported that ECM could lead to sorafenib resistance of hepatocellular carcinoma by activation of integrin-JNK signaling [10]. Besides, increased ECM stiffness in liver metastases of colorectal cancer promoted resistance to bevacizumab [11]. In our study, we demonstrated enrichment of ECM and collagen-related pathways in *ASPSCR1-TFE3* rRCC, which might be the reason why this fusion subtype responded poorly to anti-angiogenic therapy, despite higher angiogenesis activity. In addition, ECM and collagen have vital immune modulatory functions within the tumor microenvironment [12]. We also found that ECM and collagen-related signaling correlated positively with expression level of immune checkpoints, suggesting the potential application of ICI.

Aberrant angiogenesis is a hallmark of cancer, and contributes to the immune evasion of solid tumors [13, 14]. Hence, targeting the tumor vasculature to induce vessel normalization can provide a promising strategy to optimize the efficacy of immunotherapy [15]. Previous studies have shown limited therapeutic outcomes of ICI monotherapy or doublet ICI combination therapy in rRCC [5, 6]. In our study, we observed favorable efficacy of ICI plus TKI only in *ASPSCR1-TFE3* rRCC, probably through the synergistic effect between anti-angiogenic agents and immunotherapy. Moreover, compared to non-*ASPSCR1-TFE3* rRCC, increased immune-related signaling were identified in *ASPSCR1-TFE3* rRCC, which indicated a more inflamed immune microenvironment, and might further explain the efficacy of ICI based combination therapy. Given that other *TFE3*-rRCC subtypes responded poorly to ICI, further researches should be performed to explore the molecular mechanisms of these subtypes, and to propose pointed therapeutic strategy, accordingly. These findings mentioned above undoubtedly implied the importance of identifying fusion partner for every *TFE3*-rRCC patients in clinics.

This study has several limitations. Despite being the largest retrospective cohort of metastatic *TFE3*-rRCC, the small sample size due to the rarity of *TFE3*-rRCC may still limit the interpretation of the findings. Additionally, this was a single-center study thus selection bias is unavoidable. Further multi-center studies with larger sizes are required to shed further light on the development of effective therapies and selection strategies for patients suffering from this rare tumor.

## Conclusion

In conclusion, the current study represents the largest cohort comparing treatment outcomes and investigating molecular correlates of metastatic *TFE3*-rRCC. By combination of clinical data and omics analysis, we demonstrated that ICI based combination therapy was an effective first-line treatment option for metastatic *ASPSCR1-TFE3* rRCC patients. Regarding with other fusion subtypes, further studies should be performed to explore the molecular mechanisms, in order to propose pointed therapeutic strategy accordingly.

### Electronic supplementary material

Below is the link to the electronic supplementary material.

Supplementary Material 1

## Data Availability

Data is available on reasonable request.

## Abbreviations

TFE3-rRCC: TFE3-rearranged Renal Cell Carcinoma
RCC: Renal Cell Carcinoma
PFS: Progression-free Survival
ORR: Objective Response Rate
ICI: Immune Checkpoint Inhibitor
TKI: Tyrosine Kinase Inhibitor
ccRCC: Clear Cell Renal Cell Carcinoma
mTORi: Mammalian Target of Rapamycin Inhibitor
FISH: Fluorescence in situ Hybridization
ISUP: International Society of Urological Pathology
IMDC: International Metastatic RCC Database Consortium
RECIST: Response Evaluation Criteria in Solid Tumors
CR: Complete Response
PR: Partial Response
SD: Stable Disease
DCR: Disease Control Rate
OS: Overall Survival
FFPE: Formalin-fixed Paraffin-embedded
DEGs: Differential Expressed Genes
GSEA: Gene Set Enrichment Analysis
ssGSEA: Single Sample Gene Set Enrichment Analysis
RN: Radical Nephrectomy
NSS: Nephron-
CN: Cytoreductive Nephrectomy
AE: Adverse Event
ECM: Extracellular Matrix
