# Supplementary material for: Patients with ASPSCR1-TFE3 fusion achieve better response to ICI based combination therapy among TFE3-rearranged renal cell carcinoma
Source: Mol Cancer. 2024 Jun 26;23:132. doi: 10.1186/s12943-024-02044-5 (PMC11200839; doi:10.1186/s12943-024-02044-5)
Supplement: Supplementary file 1 — Supplementary Material 1 [file 12943_2024_2044_MOESM1_ESM.docx]

**Patients with *ASPSCR1-TFE3* fusion achieve better response to ICI based combination therapy among *TFE3*-rearranged renal cell carcinoma**

**Supplementary Material**

**Patients and methods**

**Study design and population**

This study is based on a retrospective, single-center, prospectively maintained database of patients with *TFE3*-rRCC. All suspicious RCC cases were referred for pathologic consultation at West China Hospital of Sichuan University, Chengdu, China. *TFE3*-rRCC was diagnosed according to immunohistochemistry evidence of positive TFE3 using commercially available primary anti-TFE3 (clone MRQ-37, 1:100, MXB biotechnologies, Fujian, China), and further confirmation of break-apart fluorescence in situ hybridization (FISH) assay by two experienced urological pathologists (N.C. and X.X.Y). Metastatic disease was confirmed by enhanced computed tomography and bone scintigraphy. Patients diagnosed with metastatic *TFE3*-rRCC and receiving systemic treatment were identified and enrolled for analysis. The study was conducted in accordance with the Declaration of Helsinki and was approved by the Ethics Committee of West China Hospital of Sichuan University. Informed consent was obtained from all patients.

**Data collection and outcomes**

Baseline demographic and clinicopathologic data were collected by individual chart review, including age at diagnosis, gender, nephrectomy status, primary tumor size, TNM stage, International Society of Urological Pathology (ISUP) grade, time of metastasis, number of metastatic organs, metastatic sites, International Metastatic RCC Database Consortium (IMDC) risk score, fusion partner, and systemic treatment type. Tumor response was assessed using Response Evaluation Criteria in Solid Tumors (RECIST) version 1.1, objective response rate (ORR) was defined as complete response (CR) + partial response (PR), disease control rate (DCR) was defined as ORR + stable disease (SD). Progression-free survival (PFS) was defined from the start of systemic therapy to disease progression or death. Overall survival (OS) was defined from the start of systemic therapy to death from any cause.

**Gene expression analysis and gene signatures**

Total RNA was isolated from formalin-fixed paraffin-embedded (FFPE) samples using the Qiagen RNeasy FFPE Kit (73504, Qiagen, Hilden, Germany) according to the manufacturer’s instructions. Gene expression profiles of tumors were generated for 32 patients using RNA-seq as previously described^1^. Fusion gene supported by at least two reads were selected. Differentially, expressed genes (DEGs) were determined using the R package “limma" with a cutoff p-value < 0.05^2^. For gene set analysis, hallmark gene sets, Gene Ontology gene sets and KEGG gene sets from MSigDB database were collected^3^. Gene set enrichment analysis (GSEA) was conducted using the GSEA software version 4.2.1. The signature score for each sample was calculated using single-sample GSEA (ssGSEA). Infiltration level of cells was calculated using CIBERSORT algorithm^4^.

**Statistical analysis**

Continuous variables were described using median and range, and categorical variables were summarized by their percentages. All comparisons for continuous variables were performed using the Student’s t-test for two groups. Survival outcomes were estimated by the Kaplan-Meier method and the difference was tested using log-rank. Cox proportional hazards regression was used to determine the independent predictor. All clinicopathologic parameters at P < 0.05 were then further tested on multivariate Cox regression. All analyses were performed using R software v.4.1.1 (R Foundation for Statistical Computing, Vienna, Austria) and SPSS v.26.0 (SPSS Inc, Chicago, IL, USA). A P < 0.05 was considered statistically significant.

**Reference**

1. Sun, G. *et al.* Integrated exome and RNA sequencing of TFE3-translocation renal cell carcinoma. *Nat Commun* **12**, 5262 (2021).

2. Ritchie, M. E. *et al.* limma powers differential expression analyses for RNA-sequencing and microarray studies. *Nucleic Acids Res* **43**, e47 (2015).

3. Liberzon, A. *et al.* Molecular signatures database (MSigDB) 3.0. *Bioinformatics* **27**, 1739–1740 (2011).

4. Chen, B., Khodadoust, M. S., Liu, C. L., Newman, A. M. & Alizadeh, A. A. Profiling Tumor Infiltrating Immune Cells with CIBERSORT. *Methods Mol Biol* **1711**, 243–259 (2018).

**Supplementary Figures**


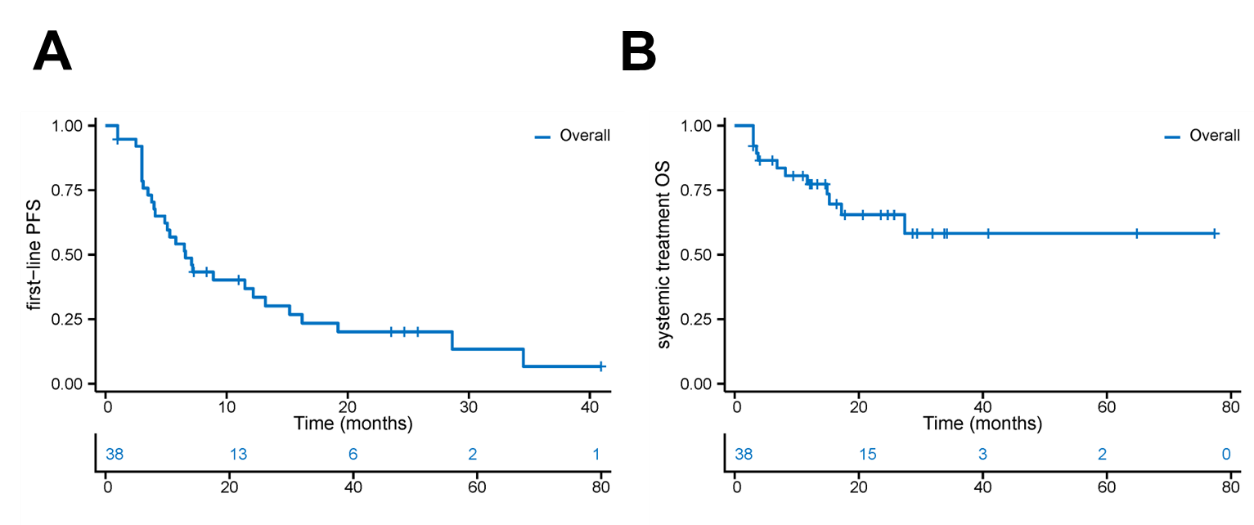


**Supplementary Figure 1.** First-line progression-free survival (PFS) and overall survival (OS) of all metastatic *TFE3*-rRCC patients receiving systemic treatment.

**A** First-line PFS of all metastatic *TFE3*-rRCC patients receiving systemic treatment. **B** OS of all metastatic *TFE3*-rRCC patients receiving systemic treatment.

**
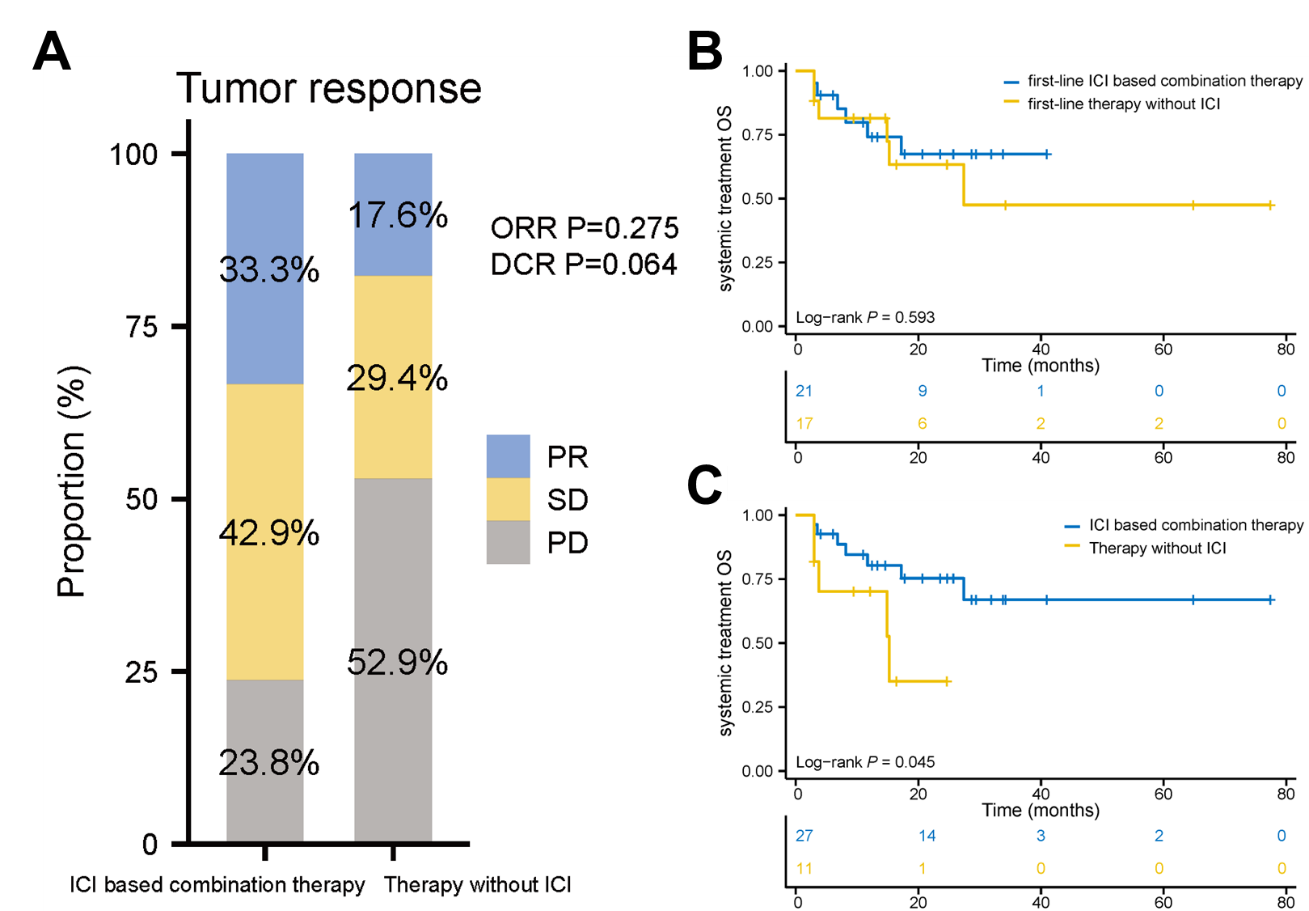
**

**Supplementary Figure 2.** Treatment outcomes between patients treated with ICI based combination therapy and therapy without ICI.

**A** Tumor response between patients receiving ICI based combination therapy and therapy without ICI at first-line. **B** OS between patients receiving ICI based combination therapy and therapy without ICI at first-line. **C** OS between patients receiving ICI based combination therapy at first or subsequent line and patients never receiving ICI during the treatment history.


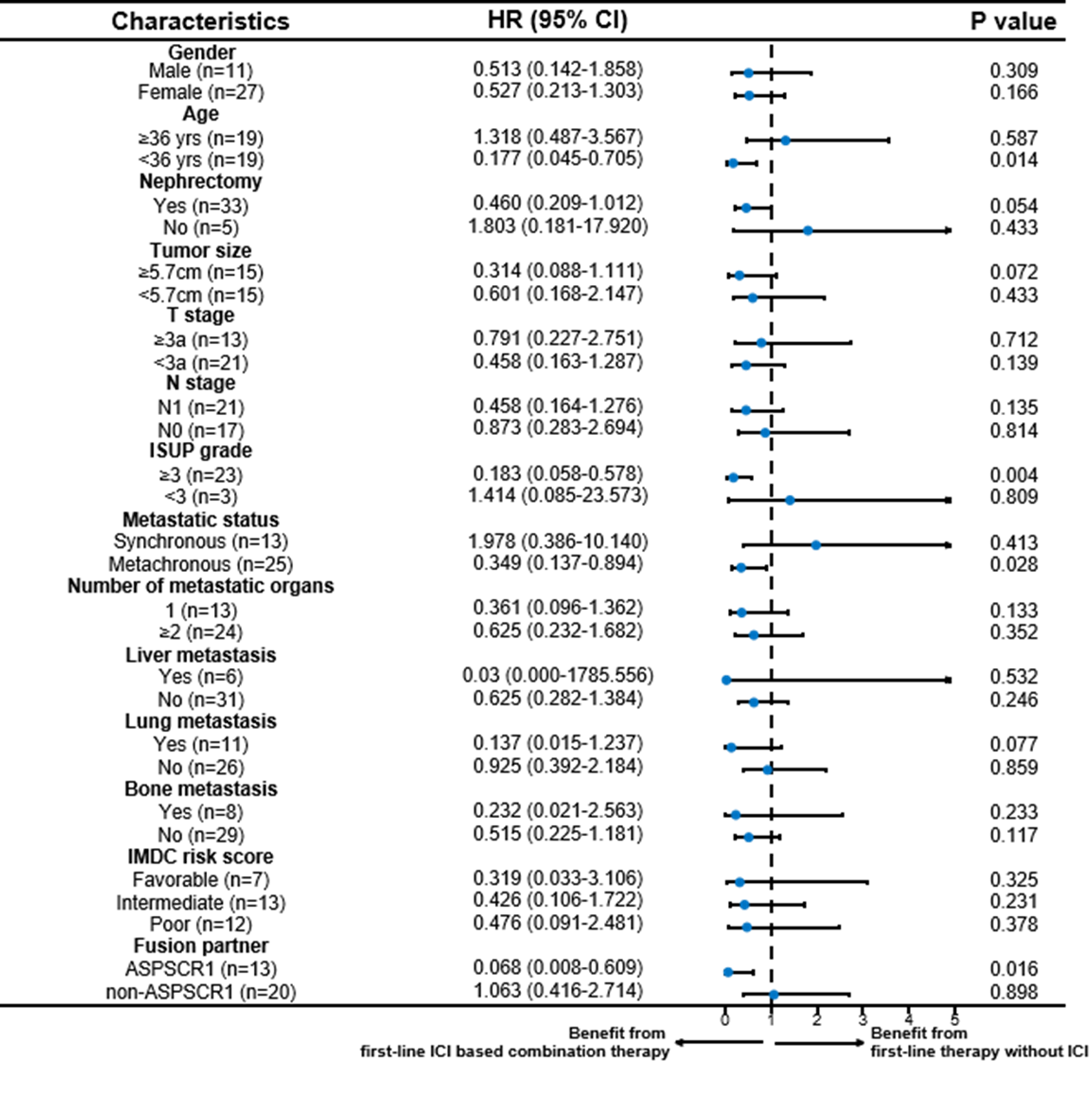


**Supplementary Figure 3.** Forest plot showing subgroups that could benefit from first-line ICI based combination therapy. HR<1 indicates better PFS receiving first-line ICI based combination therapy. HR>1 indicates better PFS receiving first-line therapy without ICI. ISUP, International Society of Urological Pathology. IMDC, International Metastatic RCC Database Consortium.


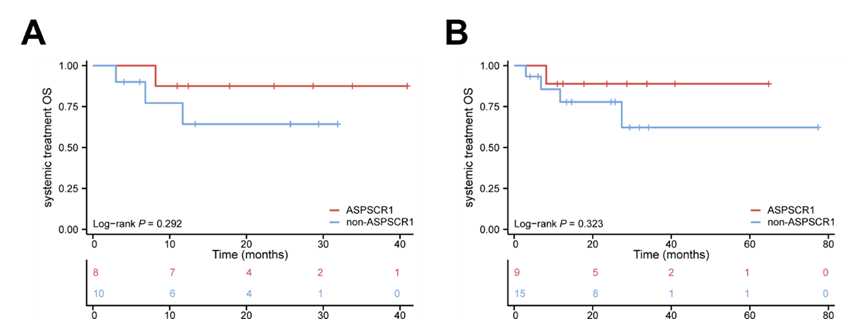


**Supplementary Figure 4.** OS between patients with *ASPSCR1-TFE3* rRCC and non-*ASPSCR1-TFE3* rRCC when receiving ICI-based combination therapy.

**A** OS between patients with *ASPSCR1-TFE3* rRCC and non-*ASPSCR1-TFE3* rRCC when receiving first-line ICI-based combination therapy. **B** OS between patients with *ASPSCR1-TFE3* rRCC and non-*ASPSCR1-TFE3* rRCC when receiving ICI based combination therapy at first or subsequent line.


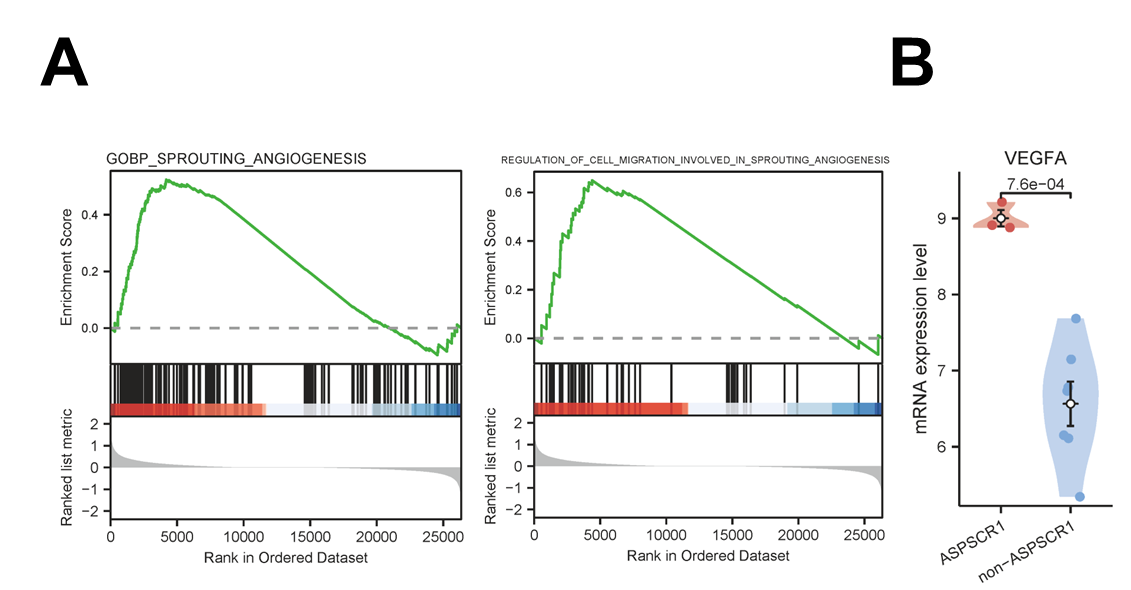


**Supplementary Figure 5.** Enrichment of angiogenesis signaling pathways in *ASCSCR1-TFE3* rRCC validated by IMmotion151 cohort.

**A** Enrichment of angiogenesis signaling pathways in *ASPSCR1-TFE3* rRCC revealed by gene set enrichment analysis in IMmotion151 cohort. **B** The mRNA expression level of *VEGFA* between *ASPSCR1-TFE3* rRCC and non-*ASPSCR1-TFE3* rRCC in IMmotion151 cohort.


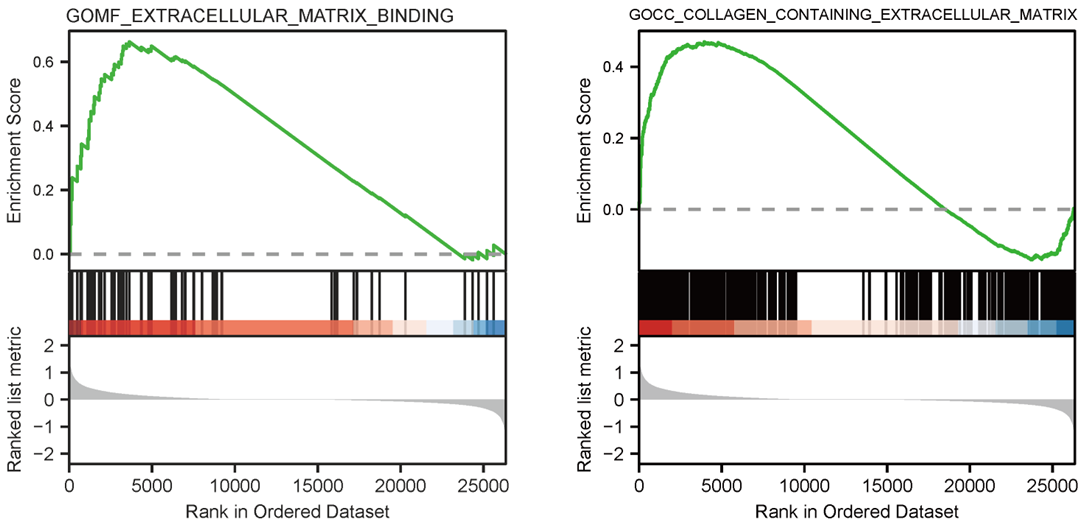


**Supplementary Figure 6.** Enrichment of ECM and collagen-related pathways in *ASPSCR1-TFE3* rRCC revealed by GSEA in IMmotion151 cohort.


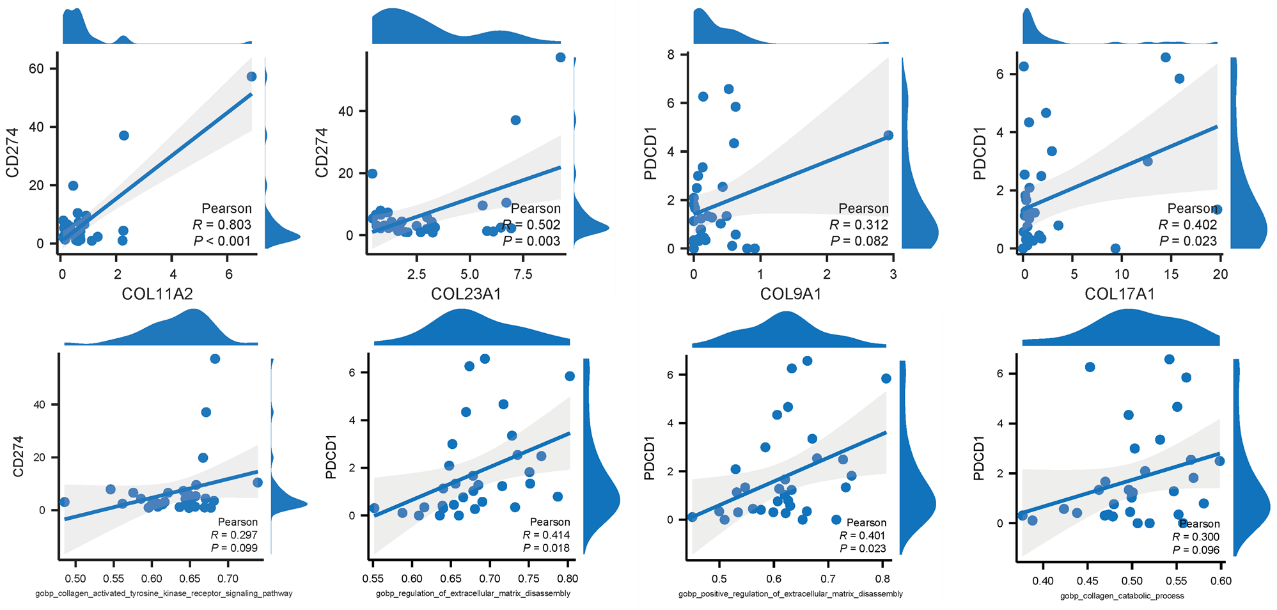


**Supplementary Figure 7.** Correlations between expression level of collagen-related genes, activities of ECM and collagen-related pathways and expression level of immune checkpoints (*CD274*, *PDCD1*) in our cohort.


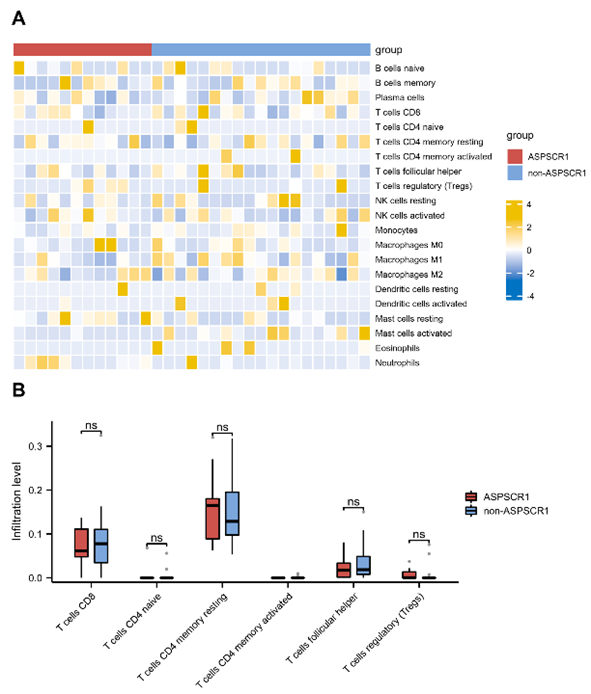


**Supplementary Figure 8.** Infiltration level of different cells between *ASPSCR1-TFE3* rRCC and non-*ASPSCR1-TFE3* rRCC using CIBERSORT algorithm.

**A** Heatmap showing infiltration level of different cells between *ASPSCR1-TFE3* rRCC and non-*ASPSCR1-TFE3* rRCC using CIBERSORT algorithm. **B** Infiltration level of different T cells between *ASPSCR1-TFE3* rRCC and non-*ASPSCR1-TFE3* rRCC using CIBERSORT algorithm.


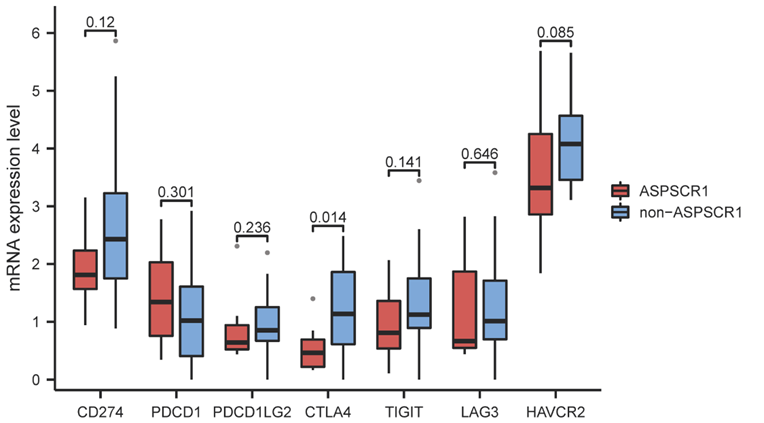


**Supplementary Figure 9.** Expression level of immune checkpoint molecules between *ASPSCR1-TFE3* rRCC and non-*ASPSCR1-TFE3* rRCC.

**Supplementary Tables**

**Supplementary Table 1** Baseline characteristics of patients with metastatic *TFE3*-rRCC.

| **Characteristics** | **n (%)** |
| --- | --- |
| No. of patients | 38 |
| Age (yr), median (range) | 36 (11-70) |
| Male-to-female ratio | 1:2.5 |
| Nephrectomy (%) |  |
| RN | 19 (50.0) |
| NSS | 6 (15.8) |
| CN | 8 (21.1) |
| No | 5 (13.2) |
| Tumor size (cm), median (range) | 5.7 (2.2-19.4) |
| T stage |  |
| <T3a | 21 (55.3) |
| ≥T3a | 13 (34.2) |
| Tx | 4 (10.5) |
| N stage |  |
| N0 | 17 (44.7) |
| N1 | 21 (55.3) |
| ISUP grade (%) |  |
| 2 | 3 (7.9) |
| 3 | 21 (55.3) |
| 4 | 2 (5.3) |
| NA | 12 (31.6) |
| Fusion partner (%) |  |
| *ASPSCR1* | 13 (34.2) |
| *NONO* | 6 (15.8) |
| *PRCC* | 5 (13.2) |
| *MED15* | 3 (7.9) |
| *SFPQ* | 2 (5.3) |
| *U2AF2* | 2 (5.3) |
| *ARID1B* | 1 (2.6) |
| *RBM10* | 1 (2.6) |
| NA | 5 (13.2) |
| Metastatic status (%) |  |
| Synchronous | 13 (34.2) |
| Metachronous | 25 (65.8) |
| Number of metastatic organs (%) |  |
| 1 | 13 (34.2) |
| ≥2 | 24 (63.2) |
| NA | 1 (2.6) |
| Metastatic sites at diagnosis (%) |  |
| Retroperitoneal lymph node | 23 (60.5) |
| Lung | 11 (28.9) |
| Bone | 8 (21.1) |
| Liver | 6 (15.8) |
| Distal lymph node | 5 (13.2) |
| IMDC risk (%) |  |
| Favorable | 7 (18.4) |
| Intermediate | 13 (34.2) |
| Poor | 12 (31.6) |
| NA | 6 (15.8) |

*TFE3*-rRCC, *TFE3*-rearranged renal cell carcinoma;

RN, radical nephrectomy; NSS, nephron-sparing surgery; CN, cytoreductive nephrectomy;

ISUP, International Society of Urological Pathology; NA, not available;

IMDC, International Metastatic RCC Database Consortium.

**Supplementary Table 2** Adverse events that occurred during first-line treatment.

| **First-line regime** | **All (n=38)** | | **First-line ICI based combination therapy (n=21)** | | **First-line therapy without ICI (n=17)** | |
| --- | --- | --- | --- | --- | --- | --- |
| Adverse event | Any grade, N (%) | Grade ≥3, N (%) | Any grade, N (%) | Grade ≥3, N (%) | Any grade, N (%) | Grade ≥3, N (%) |
| Any event | 38 (100%) | 9 (24%) | 21 (100%) | 6(29%) | 17 (100%) | 3(18%) |
| Palmar–plantar erythrodysesthesia syndrome | 20 (53%) | 1 (3%) | 9 (43%) | 0 (0%) | 11 (65%) | 1 (6%) |
| Proteinuria | 15 (39%) | 1 (3%) | 8 (38%) | 0 (0%) | 7 (41%) | 1 (6%) |
| Increased blood creatinine | 14 (37%) | 0 (0%) | 11 (52%) | 0 (0%) | 3 (18%) | 0 (0%) |
| Increased blood lactate dehydrogenase | 13 (34%) | 0 (0%) | 7 (33%) | 0 (0%) | 6 (35%) | 0 (0%) |
| Increased aspartate aminotransferase | 13 (34%) | 1 (3%) | 10 (48%) | 1 (5%) | 3 (18%) | 0 (0%) |
| Hypothyroidism | 11 (29%) | 0 (0%) | 9 (43%) | 0 (0%) | 2 (12%) | 0 (0%) |
| Hematuria | 11 (29%) | 0 (0%) | 6 (29%) | 0 (0%) | 5 (29%) | 0 (0%) |
| Anemia | 11 (29%) | 0 (0%) | 7 (33%) | 0 (0%) | 4 (24%) | 0 (0%) |
| Increased triglyceride | 9 (24%) | 0 (0%) | 8 (38%) | 0 (0%) | 1 (6%) | 0 (0%) |
| Diarrhea | 8 (21%) | 0 (0%) | 5 (24%) | 0 (0%) | 3 (18%) | 0 (0%) |
| Increased transglutaminase | 8 (21%) | 0 (0%) | 5 (24%) | 1 (5%) | 3 (18%) | 0 (0%) |
| Hyperuricemia | 8 (21%) | 0 (0%) | 8 (38%) | 0 (0%) | 2 (12%) | 0 (0%) |
| Decreased lymphocyte count | 8 (21%) | 0 (0%) | 7 (33%) | 0 (0%) | 1 (6%) | 0 (0%) |
| Increased alanine transaminase | 8 (21%) | 0 (0%) | 6 (29%) | 0 (0%) | 2 (12%) | 0 (0%) |
| Adrenal insufficiency | 7 (18%) | 0 (0%) | 6 (29%) | 0 (0%) | 1 (6%) | 0 (0%) |
| Hypertension | 6 (16%) | 2 (5%) | 4 (19%) | 1 (5%) | 2 (12%) | 1 (6%) |
| Abdominal pain | 6 (16%) | 0 (0%) | 3 (14%) | 0 (0%) | 3 (18%) | 0 (0%) |
| Hypercholesteremia | 6 (16%) | 0 (0%) | 5 (24%) | 0 (0%) | 1 (6%) | 0 (0%) |
| Increased leukocyte count | 6 (16%) | 0 (0%) | 6 (29%) | 0 (0%) | 0 (0%) | 0 (0%) |
| Hypoalbuminemia | 6 (16%) | 0 (0%) | 4 (19%) | 0 (0%) | 2 (12%) | 0 (0%) |
| Hyponatremia | 6 (16%) | 0 (0%) | 5 (24%) | 0 (0%) | 1 (6%) | 0 (0%) |
| Hyperglycemia | 6 (16%) | 1 (3%) | 5 (24%) | 1 (5%) | 1 (6%) | 0 (0%) |
| Nausea | 5 (13%) | 0 (0%) | 4 (19%) | 0 (0%) | 1 (6%) | 0 (0%) |
| Dental ulcer | 3 (8%) | 0 (0%) | 2 (10%) | 0 (0%) | 1 (6%) | 0 (0%) |
| Decreased blood platelet count | 3 (8%) | 0 (0%) | 0 (0%) | 0 (0%) | 3 (18%) | 0 (0%) |
| Increased alkaline phosphatase | 3 (8%) | 0 (0%) | 3 (14%) | 0 (0%) | 0 (0%) | 0 (0%) |
| Throat pain | 2 (5%) | 0 (0%) | 2 (10%) | 0 (0%) | 0 (0%) | 0 (0%) |
| Hoarseness | 2 (5%) | 0 (0%) | 2 (10%) | 0 (0%) | 0 (0%) | 0 (0%) |
| Vomiting | 2 (5%) | 0 (0%) | 1 (5%) | 0 (0%) | 1 (6%) | 0 (0%) |
| Dizziness | 2 (5%) | 0 (0%) | 1 (5%) | 0 (0%) | 1 (6%) | 0 (0%) |
| Increased cardiac troponin T | 2 (5%) | 1 (3%) | 2 (10%) | 1 (5%) | 0 (0%) | 0 (0%) |
| Hypomagnesemia | 2 (5%) | 0 (0%) | 2 (10%) | 0 (0%) | 0 (0%) | 0 (0%) |
| Hypocalcemia | 2 (5%) | 0 (0%) | 1 (5%) | 0 (0%) | 1 (6%) | 0 (0%) |
| Cough | 1 (3%) | 0 (0%) | 1 (5%) | 0 (0%) | 0 (0%) | 0 (0%) |
| Abdominal distension | 1 (3%) | 0 (0%) | 1 (5%) | 0 (0%) | 0 (0%) | 0 (0%) |
| Alopecia | 1 (3%) | 0 (0%) | 0 (0%) | 0 (0%) | 1 (6%) | 0 (0%) |
| Ascites | 1 (3%) | 0 (0%) | 1 (5%) | 0 (0%) | 0 (0%) | 0 (0%) |
| Intestinal fistula | 1 (3%) | 1 (3%) | 1 (5%) | 1 (5%) | 0 (0%) | 0 (0%) |
| Decreased level of consciousness | 1 (3%) | 1 (3%) | 1 (5%) | 1 (5%) | 0 (0%) | 0 (0%) |
| Increased blood bilirubin | 1 (3%) | 0 (0%) | 0 (0%) | 0 (0%) | 1 (6%) | 0 (0%) |
| Hypophosphatemia | 1 (3%) | 0 (0%) | 0 (0%) | 0 (0%) | 1 (6%) | 0 (0%) |
| Prolonged APTT | 1 (3%) | 0 (0%) | 1 (5%) | 0 (0%) | 0 (0%) | 0 (0%) |
| Hypercalcemia | 1 (3%) | 0 (0%) | 0 (0%) | 0 (0%) | 1 (6%) | 0 (0%) |
| Hypermagnesemia | 1 (3%) | 0 (0%) | 0 (0%) | 0 (0%) | 1 (6%) | 0 (0%) |

ICI, immune checkpoint inhibitor; APTT, activated partial thromboplastin time.

**Supplementary Table 3** Univariate and multivariate Cox regression analysis of progression-free survival in patients receiving first-line ICI based combination therapy.

| **Characteristics** | **Comparisons** | **Univariate analysis** | | **Multivariate analysis** | |
| --- | --- | --- | --- | --- | --- |
|  |  | **HR (95% CI)** | **P value** | **HR (95% CI)** | **P value** |
| Age | ≥ 36 yrs vs. < 36 yrs | 2.027 (0.674-6.093) | 0.208 |  |  |
| Gender | Male vs. Female | 2.576 (0.805-8.242) | 0.111 |  |  |
| Nephrectomy | Yes vs. No | 0.573 (0.154-2.130) | 0.406 |  |  |
| T stage | ≥T3a vs. <T3a | 2.432 (0.723-8.180) | 0.151 |  |  |
| N stage | N1 vs. N0 | 0.590 (0.188-1.848) | 0.365 |  |  |
| ISUP grade | ≥3 vs. <3 | 0.406 (0.047-3.489) | 0.412 |  |  |
| Metachronous status | Metachronous vs. Synchronous | 0.309 (0.095-1.001) | 0.050 | 0.476 (0.090-2.534) | 0.384 |
| Number of metastatic organs | ≥2 vs. 1 | 0.712 (0.248-2.041) | 0.527 |  |  |
| Liver metastasis | Yes vs. No | 0.045 (0.000-5969.925) | 0.606 |  |  |
| Lung metastasis | Yes vs. No | 0.551 (0.150-2.019) | 0.368 |  |  |
| Bone metastasis | Yes vs. No | 4.096 (1.061-15.818) | 0.041 | 0.271 (0.045-1.627) | 0.153 |
| IMDC risk score | Intermediate/Poor vs. Favorable | 5.615 (0.693-45.457) | 0.106 |  |  |
| Fusion partner | *ASPSCR1* vs. non-*ASPSCR1* | 0.160 (0.033-0.763) | 0.022 | 0.184 (0.035-0.967) | 0.045 |

HR, harzard ratio; CI, confidence interval; ISUP, International Society of Urological Pathology; IMDC, International Metastatic RCC Database Consortium.

**Supplementary Table 4** Survival outcomes for *TFE3*-rRCC patients with each fusion subtype receiving different first-line systemic treatments.

| **First-line treatment** | **Fusion partner** | **Number of patients** | **mPFS (months)** | **ORR** |
| --- | --- | --- | --- | --- |
| **ICI based combination therapy** | ***ASPSCR1*** | 8 | NR | 62.5% |
|  | ***NONO*** | 4 | 6.6 | 0.0% |
|  | ***PRCC*** | 2 | 4.9 | 0.0% |
|  | **Others** | 4 | 4.1 | 25.0% |
|  | **non-*ASPSCR1*** | 10 | 6.5 | 10.0% |
| **Therapy without ICI** | ***ASPSCR1*** | 5 | 3.0 | 0.0% |
|  | ***NONO*** | 2 | 3.0 | 50.0% |
|  | ***PRCC*** | 3 | 8.9 | 66.7% |
|  | **Others** | 5 | 5.1 | 0.0% |
|  | **non-*ASPSCR1*** | 10 | 5.1 | 30.0% |

*TFE3*-rRCC, *TFE3*-rearranged renal cell carcinoma;

mPFS, median progression-free survival; ORR, objective response rate;

ICI, immune checkpoint inhibitor.

**Supplementary Table 5.** Identification of partner genes in 10 out of 12 patients with *TFE3* fusion from IMmotion151 trial.

| **Symbol** | **Fusion** | **Arm** | **PFS time** | **PFS status** | **Tumor response** |
| --- | --- | --- | --- | --- | --- |
| EA-0d2902fb00 | PRCC | SUN | 2.83 | Dead | PD |
| EA-11ad54998e | PRCC | ATEZO+BEV | 13.96 | Censored | SD |
| EA-173d2828ca | PRCC | ATEZO+BEV | 15.77 | Dead | SD |
| EA-4770aabbaf | ASPSCR1 | ATEZO+BEV | 18 | Censored | PR |
| EA-504761f9a6 | SFPQ | SUN | 4.73 | Dead | SD |
| EA-789aa25ce7 | RBM10 | SUN | 0.89 | Dead | PD |
| EA-ba69a9f969 | NONO | ATEZO+BEV | 3.78 | Dead | SD |
| EA-ea91444a0a | ASPSCR1 | ATEZO+BEV | 11.24 | Dead | SD |
| EA-ee0715d5d6 | RBM10 | SUN | 4.14 | Dead | SD |
| EA-fd72b64a6f | ASPSCR1 | SUN | 6.83 | Dead | SD |
